# Molecular docking and proteomics reveals the synergistic antibacterial mechanism of theaflavin with β-lactam antibiotics against MRSA

**DOI:** 10.3389/fmicb.2022.993430

**Published:** 2022-11-14

**Authors:** Shuhan Guan, Ling Zhong, Hangqian Yu, Li Wang, Yajing Jin, Jingyu Liu, Hua Xiang, Hao Yu, Lin Wang, Dacheng Wang

**Affiliations:** ^1^College of Animal Science, Jilin University, Changchun, China; ^2^Changchun University of Chinese Medicine, Changchun, China; ^3^College of Animal Medicine, Jilin Agricultural University, Changchun, China; ^4^State Key Laboratory for Zoonotic Diseases, Institute of Zoonosis, College of Veterinary Medicine, Jilin University, Changchun, China

**Keywords:** MRSA, theaflavin, β-lactam antibiotics, molecular docking, synergistic combinations

## Abstract

Recurrent epidemics of methicillin-resistant *Staphylococcus aureus* (*S. aureus*) (MRSA) have illustrated that the effectiveness of antibiotics in clinical application is rapidly fading. A feasible approach is to combine natural products with existing antibiotics to achieve an antibacterial effect. In this molecular docking study, we found that theaflavin (TF) preferentially binds the allosteric site of penicillin-binding protein 2a (PBP2a), inducing the PBP2a active site to open, which is convenient for β-lactam antibiotics to treat MRSA infection, instead of directly exerting antibacterial activity at the active site. Subsequent TMT-labeled proteomics analysis showed that TF treatment did not significantly change the landscape of the *S. aureus* USA300 proteome. Checkerboard dilution tests and kill curve assays were performed to validate the synergistic effect of TF and ceftiofur, and the fractional inhibitory concentration index (FICI) was 0.1875. The antibacterial effect of TF combined with ceftiofur was better than that of single-drug treatment *in vitro*. In addition, TF effectively enhanced the activity of ceftiofur in a mouse model of MRSA-induced pneumonia. Our findings provide a potential therapeutic strategy to combine existing antibiotics with natural products to resolve the prevalent infections of multidrug-resistant pathogens.

## Introduction

*Staphylococcus aureus* (*S. aureus*) is the leading cause of hospital and community-associated infections worldwide, with high morbidity and mortality (Tacconelli et al., 2018), especially with the emergence and rapid spread of methicillin-resistant *S. aureus* (MRSA), which makes bacterial infections more difficult to cure. Approximately, 60–70% of *S. aureus* infections are caused by MRSA, which can lead to various infectious diseases, including pneumonia, endocarditis, skin abscesses, and joint infections (Kluytmans et al., 1997). MRSA is associated with higher mortality due to resistance to multiple clinically used β-lactam antibiotics (Taubes, 2008; Boucher et al., 2009). Currently, the antibiotics of choice for treating MRSA infections are ceftaroline (Natalia et al., 2012), daptomycin (French, 2006), vancomycin (Walsh, 1999), and linezolid (Novy et al., 2011). However, in the past 20 years, the development of new antibiotics has not kept up with the emergence of drug resistance. There have been numerous reports of MRSA strains becoming resistant to last-line antibiotics (Desbois et al., 2010). In addition, antibiotic therapy exerts survival pressure on pathogenic bacteria, prompting the development of drug resistance. To this end, the combination of the two drugs or more drugs seem to be a feasible method to deal with the infection of multidrug-resistant pathogens. In general, there are three forms of drug association: synergetic, additive and antagonistic (Liu et al., 2016). Among them, synergistic effect is the most ideal, because it can be used at a lower dose, which not only reduces the cost and toxicity, but also slows down the development of antibiotic resistance (Jorge et al., 2017). Many studies have reported synergistic effects from combinations of natural products and antibiotics (Adwan and Mhanna, 2009; Aiyegoro et al., 2009).The synergistic effect of the combination of two agents has the advantages of expanding the antimicrobial spectrum, reducing toxicity and preventing the emergence of multidrug-resistant bacteria, which has become a new strategy against the current epidemic pathogens (Sanhueza et al., 2017).

Resistance in MRSA is produced by obtaining a mobile genetic element, staphylococcal chromosomal cassette mec (SCCmec), which carries penicillin-binding protein 2a (PBP2a, encoded by the *mecA* gene) with transpeptidase activity (Jensen and Lyon, 2009). Unlike other penicillin-binding proteins (PBPs), PBP2a has a closed active center conformation, a structural feature that results in a low affinity of PBP2a to almost all β-lactam antibiotics, allowing bacteria to maintain biosynthesis of cell walls in the presence of antibiotics (Lim et al., 2002). However, the active center conformation of PBP2a can be regulated by an allosteric site: when the allosteric site is triggered, the originally closed active center transforms into an open state that is easily accessible by β-lactam antibiotics (Otero et al., 2013).

Currently, some compounds have been reported to effectively inhibit MRSA as allosteric modulators of PBP2a. Ceftaroline is a fifth-generation cephalosporin that is effective against MRSA infection. Crystallographic studies showed that ceftaroline combined with the allosteric center of PBP2a, paving the way for the combination of another molecule, ceftaroline, with the active center (Otero et al., 2013). They observed that ceftaroline acylated the serine at the active site of PBP2a and that a second ceftaroline molecule bound to the noncovalent binding region 60 Å from the active center. The binding of the ceftaroline molecule at this allosteric site was translated into a series of conformational changes through a unique salt bridge interaction, resulting in a twist of the β3 strand and a shift of the α2-α3 loop near the active site, allowing the antibiotic to enter the active site for acylation. Quinazolinone is a biologically active nitrogen ring compound with a wide range of biological properties such as antibacterial, antifungal, anticonvulsant, anti-inflammatory, anti-HIV, anticancer, and analgesic activities (Jafari et al., 2016; Hameed et al., 2018). Jeshina *et al* (Janardhanan et al., 2019) reported that quinazolinone exhibited a significant synergistic bactericidal effect when used in triple combination with piperacillin and tazobactam. The X-ray crystal structure confirmed the binding of quinazolinone to the allosteric site of PBP2a, which ultimately resulted in the opening of the active center, allowing piperacillin to bind and subsequently inhibit PBP2a. However, the use of synthetic drugs is often limited due to their undesirable side effects (Dancer, 2001). With the development of anti-MRSA drugs, the therapeutic potential of natural compounds has been increasingly recognized. Some potential plant drug candidates have shown promising activities and can be further developed into clinical therapeutics (Hemaiswarya et al., 2008; Novy et al., 2011). Theaflavin (TF) is a polyphenolic compound commonly found in fermented tea that has various pharmacological effects, such as antimicrobial (Betts et al., 2013), anti-inflammatory (Kim and Joo, 2011), antioxidant (Yang et al., 2007), and antitumor (Chakrabarty et al., 2019) effects. Sato et al. (2022) reported that Theaflavin and its derivatives exert antibacterial action against *Bacillus coagulans* through adsorption to cell surface phospholipids (Sato et al., 2022). In addition, Teng et al. (2019) reported that Theaflavin-3,3′-digallate, a theaflavin derivative, increased the antibacterial activity of β-lactam antibiotics by inhibiting the activity of metallo-β-lactamases (Teng et al., 2019). Studies have shown that TF has good bacteriostatic effects, including direct antibacterial activity, synergistic antibiotic bacteriostasis, and bacterial virulence inhibition (Betts et al., 2011, 2012, 2017). Therefore, targeting allosteric sites to find allosteric regulators is an effective way to restore β-lactam antibiotic sensitivity and fight MRSA infection.

In this research, we explored the binding efficiency of TF as a ligand to the allosteric center of PBP2a through molecular docking, speculating that TF could act as an allosteric agent of PBP2a to regain the sensitivity of PBP2a to β-lactam antibiotics, thus fighting against MRSA infection. Furthermore, we determined the total protein expression differences induced by TF by a TMT-based quantitative proteomic approach to further explore its effects on MRSA. In addition, *in vitro* and *in vivo* experiments were carried out to verify that low-dose TF can produce synergistic anti-MRSA effects with a variety of β-lactam antibiotics. Our study provides a theoretical basis for the further development and application of TF as an antibacterial therapy against drug-resistant bacteria.

## Materials and methods

### Molecular docking

Molecular docking calculations were performed with Schrodinger® docking suits (Schrödinger Maestro, New York) using a virtual screening workflow. The chemical structure of theaflavin was extracted from the PubChem repository.^1^ The ligand (PBP2a) was prepared using the LigPrep tool and embedded in Schrödinger suite-Maestro. Ligand-binding sites of receptors were predicted using the SiteMap module in the Schrodinger Suite-Maestro. Five potential active sites were analyzed based on the top-ranked site scores. Docking scores are reported in kcal/mol.

### Reagents and materials

TF was purchased from Ruifensi Company (Chengdu, China). MHB and CAMHB media for bacterial culture were purchased from Haibo Biotechnology (Qingdao, China). Antibiotics: Ceftiofur, cefoxitin, ceftaroline fosamil, latamoxef, ceftazidime, cefepime, oxacillin, ampicillin and penicillin G were purchased from Meilun Biotechnology Co., Ltd. (Dalian, China).

### Bacteria and growth conditions

*Staphylococcus aureus* USA300 was purchased from the American Type Culture Collection (ATCC, Manassas, VA, United States). MRSA clinical isolates were isolated in the past 3 years and identified by 16S rRNA and quality control. *S. aureus* was cultured in MHB at 35°C and 220 rpm.

### Sample preparation for TMT-labeled proteomics analysis

Overnight culture of USA300 was diluted 1:100 in MHB media with or without 64 μg/mL TF and shaken at 35°C to an OD_600_ of 1.5. Then, the bacteria were collected by centrifugation at 4°C and 5,000 rpm for 10 min and rinsed with normal saline three times. Subsequently, bacterial precipitates were collected and rapidly frozen in liquid nitrogen. SDT lysis was used to extract bacterial protein according to the manufacturer’s instructions (Wiśniewski et al., 2009), and then the protein was quantified using a BCA kit (Beyotime, Shanghai, China). For proteomics sample preparation, the protein was digested by trypsin (1:50 w/w, Promega, Madsion, WI) at 37°C overnight to prepare peptides. Then, 100 μg peptide was taken from each group and labeled with TMT isobaric tags based on the TMT kit (Thermo Fisher Scientific, United States) instructions. Then, the nano LC–MS/MS experiment was carried out with an Orbitrap elite LC–MS/MS (Thermo Fisher Scientific, United States) as previously described (Liu et al., 2016; Ma et al., 2017). At least three biological replications were performed in each group.

### Protein identification and quantification

Proteome Discoverer software 1.4 and Mascot 2.2 were used to analyze the TMT-labeled proteomics data. The parameters for database searching were set as follows: (1) quantification type: TMT sixplex; (2) digestion: trypsin; (3) mass tolerance: 10 ppm; and (4) fragment mass tolerance: 0.1 Da. The DESeq2 package was used for normalization and differential expression analysis (Osabe et al., 2021). Adjusted *p* < 0.05 and fold change >1.5 were set as the cutoff criteria for differentially expressed proteins (DEPs). StaphNet is a functional gene network model for MRSA. The network was constructed based on the genome of *S. aureus* subspecies USA300_FPR3757, a representative MRSA strain. To understand the biological functions of candidate genes, this study mapped the DEPs to the online website StaphNet^2^ for gene ontology (GO) enrichment analysis and Kyoto Encyclopedia of Genes and Genomes (KEGG) pathway analysis. StaphNe’s web server can generate a variety of biological hypotheses, and using StaphNet can identify genes for virulence-related phenotypes of MRSA or search for new candidate genes. The use of StaphNet can be based on what has been previously described in the literature (Kim et al., 2018). A pathway-centric search engine was applied to identify potential interactions between DEPs and the data were further imported into Cytoscape to build PPI networks. A volcano plot and a bubble chart were illustrated using the R package “ggplot2” (Wickham, 2009).

### Determination of allosteric site binding affinity by fluorescent quenching

The binding affinity of TF to the allosteric site of PBP2a was investigated as previously described by Fishovitz (Fishovitz et al., 2014). The active site of PBP2a was first incubated with an excess of oxacillin at room temperature for 45 min to allow complete acylation. The unbound remaining oxacillin was removed by centrifugation using an ultrafiltration centrifuge tube. The binding of TF to the allosteric site was determined by monitoring the intrinsic fluorescence of PBP2a after being occupied at the active site.

Subsequently, the above-treated PBP2a (1 μM) was incubated for 10 min at room temperature in a buffer containing HEPES (pH 7, 25 mM) and NaCl (1 M). The emission spectra of PBP2a alone were recorded after excitation at 280 nm. The emission spectra were measured when TF was titrated into the reaction. The decrease in fluorescence emission at 330 nm was measured for each concentration of TF. The normalized difference in fluorescence versus concentration was displayed. All experiments were performed at least in triplicate.

### Real-time quantitative PCR

RT–qPCR was carried out to assess the gene transcription levels of USA300 treated with TF. First, an overnight culture of USA300 was diluted 1:100 in MHB media containing 64 μg/mL TF and shaken at 35°C to an OD_600_ of 1. Then, the bacteria were collected by centrifugation, and total RNA was extracted using a Qiagen RNeasy kit (Tiangen, Beijing, China). The concentration of RNA was measured using a NanoDrop 1,000 spectrophotometer (Thermo Fisher Scientific, United States). Subsequently, reverse transcription was carried out using PrimeScript RT MasterMix (Takara, Dalian, China), according to the manufacturer’s instructions. The primers designed in this study are listed in Supplementary Table S1, and 16S rRNA was used as the internal reference gene. cDNA was amplified using the SuperReal PreMix Plus (Tiangen, Beijing, China) on the Applied Biosystems 7,300 Real-time PCR System with the following cycle parameters: 95°C for 30 s, followed by 40 cycles of 95°C for 5 s, 60°C for 30 s, and 72°C for 30 s. All RT–qPCRs were performed with three biological replicates. The fold-changes in gene expression were measured using the 2^−ΔΔCt^ method.

### Antibacterial tests

The minimum inhibitory concentrations (MICs) of TF and other β-lactam antibiotics against USA300 were measured using a broth microdilution method as described previously (Weinstein et al., 2020). Briefly, 96-well plates containing TF or other β-lactam antibiotics were two-fold diluted in 100 μL CAMHB and mixed with 10^5^ colony-forming units (CFUs). After incubation at 37°C for 18 h, the absorbance was measured at 600 nm, and experiments were repeated with at least three biological replicates.

### Time-kill assay

A time-killing assay was carried out to determine the synergistic effect of TF with ceftiofur or ampicillin as described previously (Mun et al., 2014; López-Gavín et al., 2016). Overnight cultures of USA300 were diluted to 10^6^ colony-forming units (CFUs)/mL in 10 mL MHB medium. Subsequently, different concentrations of agents were added to the bacterial solution, including the combination group (64 μg/mL TF + 1/2 MIC of ceftiofur or ampicillin), TF group (64 μg/mL), and antibiotic group (1/2 MIC of ceftiofur or ampicillin). *S. aureus* USA300 without any agents was used as a control group. After 0, 4, 8, 12, and 24 h of incubation at 37°C with shaking, 100 μL samples were plated on MHB agar plates and incubated at 37°C until a single colony appeared. Colonies were counted at each time point, the data were expressed as log10^CFU^, and experiments were performed with three biological replicates.

### Measurement of synergy

Checkerboard susceptibility assays were performed to measure combinations of TF and β-lactam antibiotics in a 96-well plate as previously described (Novy et al., 2011; Mohammadi et al., 2017). Briefly, β-lactam antibiotics were diluted along the ordinate, whereas TF was diluted along the abscissa. The TF and antibiotics were two-fold diluted in 100 μL CAMHB and mixed with 10^5^ CFU *S. aureus* USA300. After 18 h of incubation at 37°C, the MIC was measured by a microplate reader (Tecan Infinite M200, Switzerland). The fractional inhibitory concentration index (FICI) was calculated according to the formula below (Bonapace et al., 2002).

FICI = MIC_AB_/MIC_A_ + MIC_BA/_MIC_B_ = FIC_A_ + FIC_B_

A FIC index ≤0.5 was defined as synergy, that in the range of 0.5–1 as an additive or partial synergy, that in the range of 1–2 as no synergy, and that greater than 2 as antagonistic (Si et al., 2021).

### Pneumonia model experiment

The pneumonia model was established according to a published protocol (Bubeck Wardenburg et al., 2007; Labandeira-Rey et al., 2007; Brown et al., 2009). Eight-week-old female C57BL/6 J mice were purchased from Changsheng Biotechnology (Liaoning, China). For survival experiments, bacterial cultures of USA300 (4 × 10^8^ CFUs) for each mouse were prepared and infected intranasally. After challenging for 2 h, a specific dosage of TF (40 mg/kg), ceftiofur (100 mg/kg), and the combination of TF (40 mg/kg) and ceftiofur (100 mg/kg) were administered by subcutaneous injection (*n* = 10). The drugs were administered again at 12 h intervals after infection. The mice in the control group were given the same volume of sterile saline containing 0.5% DMSO. The status of the mice was observed, and the survival rate of each group at 12 h intervals within 96 h after administration was calculated. For the lung tissue bacterial count assay and histopathological analysis, the methods of challenge and treatment were the same as previously described (*n* = 6). After infection for 24 h, mice in each group were euthanized by carbon dioxide anesthesia and then cervical dislocation.

The left lung of mice in each group was aseptically collected, weighed, homogenized in 1 mL sterile 0.9% normal saline, and then diluted on an MHB agar plate until a single colony was grown and counted. The right lung was fixed in 10% formalin and embedded in paraffin for conventional H&E staining. Finally, the sections of lung tissue were observed under a microscope and photographed.

### Ethical statement

The animal experiments in this study were conducted in full compliance with the principles of the Basel Declaration and the guidelines of the Committee on Animal Care and Use of Jilin University.

### Statistical analysis

All data are presented as the means ± standard deviation (SD). GraphPad Prism software v8.0 (GraphPad Software, United States) was used for all statistical analyses. The results were considered significantly different at a *p* value of <0.05.

## Results

### Molecular docking of TF to PBP2a of MRSA

**Figure 1:**
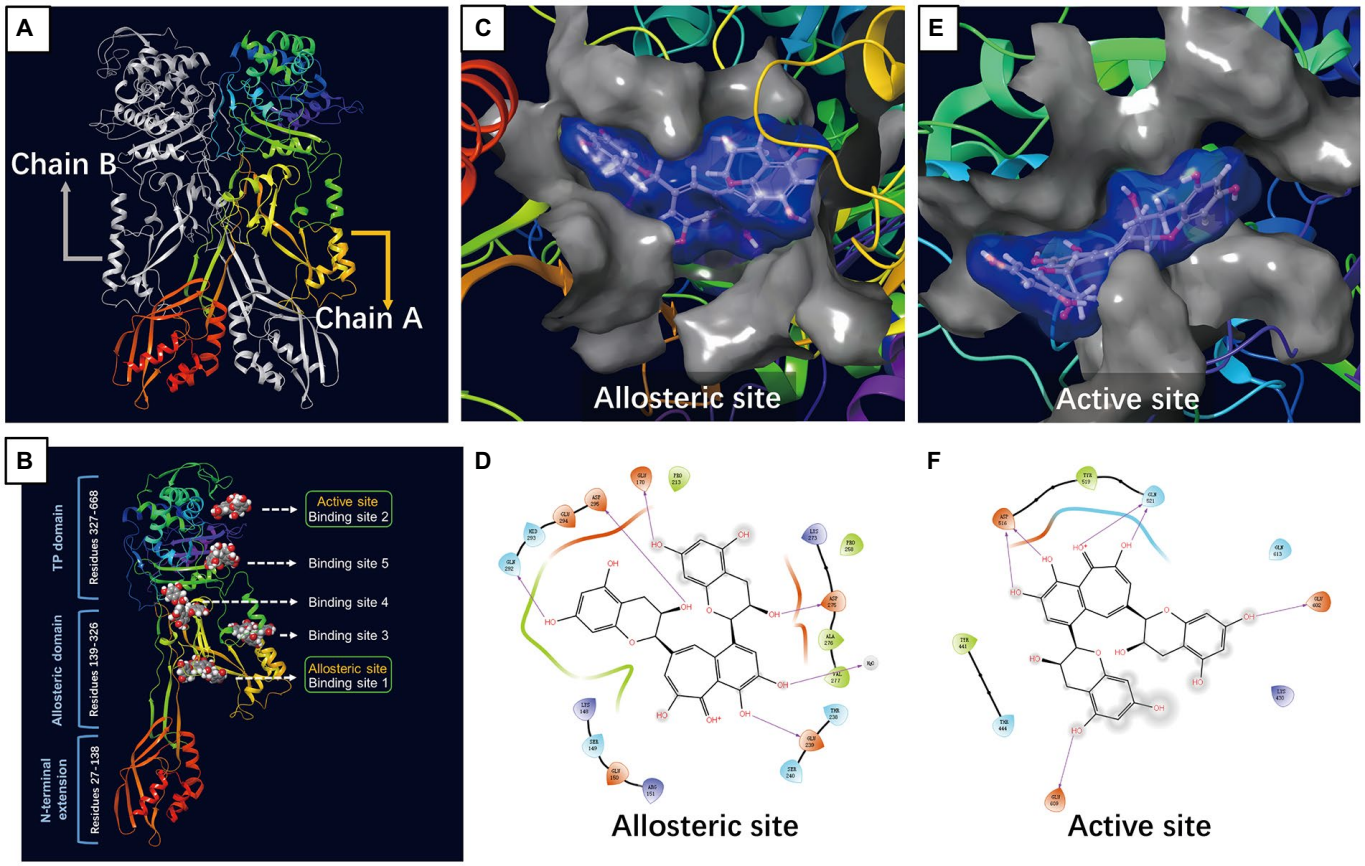
Molecular docking of TF to PBP2a of MRSA. **(A)** PBP2a protein structure (PDB: 6H5O). **(B)** The structure of PBP2a showing 5 binding sites bound to TF. **(C)** Surface representation of allosteric site bound to TF. **(D)** Amino acid residues and the nature of the interaction between TF and PBP2a at the allosteric site. **(E)** Surface representation of active site bound to TF. **(F)** Amino acid residues and the nature of the interaction between TF and PBP2a at the active site.

Molecular docking studies of the ligands to protein active sites were conducted by the advanced molecular docking program Schrodinger Maestro-11.8 version to identify the binding affinities of PBP2a with TF. We used SiteMap to identify the potential binding sites on chain A/PBP2a (PDB ID: 6H5O), as shown in Figure 1A. This led to the identification of the 5 most likely binding sites, named binding sites 1, 2, 3, 4, and 5. According to the positional information of the PBP2a structure from RCSB (such as PDB ID: 3zg5, 5m1a, 5 m18, 5 m19 and 6Q9N), we found that binding site 1 was located at the allosteric site of PBP2a, while binding site 2 was at the active site of PBP2a, and the docking results showed that TF can dock at all of the above 5 binding sites (Figure 1B). Docking scoring revealed that the lowest value for the potential energy of a docked conformer of TF to binding site 1 (allosteric site) of PBP2a was−7.218 kcal/mol, while the docking score at binding site 2 (active site) was-5.360 kcal/mol (Table 1). As observed from the 2D and 3D interactions of PBP2a-TF 01 (Figures 1C,D) and PBP2a-TF 02 (Figures 1E,F), we found that TF displayed a stronger interaction at binding site 1 (allosteric site) of PBP2a. It is estimated that TF can enhance the affinity of PBP2a for β-lactam antibiotics by binding to the allosteric site to induce the opening of the active site. Therefore, it is necessary to confirm this hypothesis by studying the synergistic antibacterial effect of TF and β-lactam antibiotics on MRSA.

**Table 1:** Energy of conformer values of PBP2a-theaflavin (PBP2a-TF).

Compound	Binding site	Docking score (kcal/mol)	Glide model (kcal/mol)
PBP2a-TF 01	Binding site 1 (allosteric site)	−7.218	−101.195
PBP2a-TF 02	Binding site 2 (active site)	−5.36	−68.895
PBP2a-TF 03	Binding site 3	−6.16	−80.772
PBP2a-TF 04	Binding site 4	−5.661	−64.011
PBP2a-TF 05	Binding site 5	−5.646	−61.202

### Proteomics profile change of *Staphylococcus aureus* treated with TF

**Figure 2:**
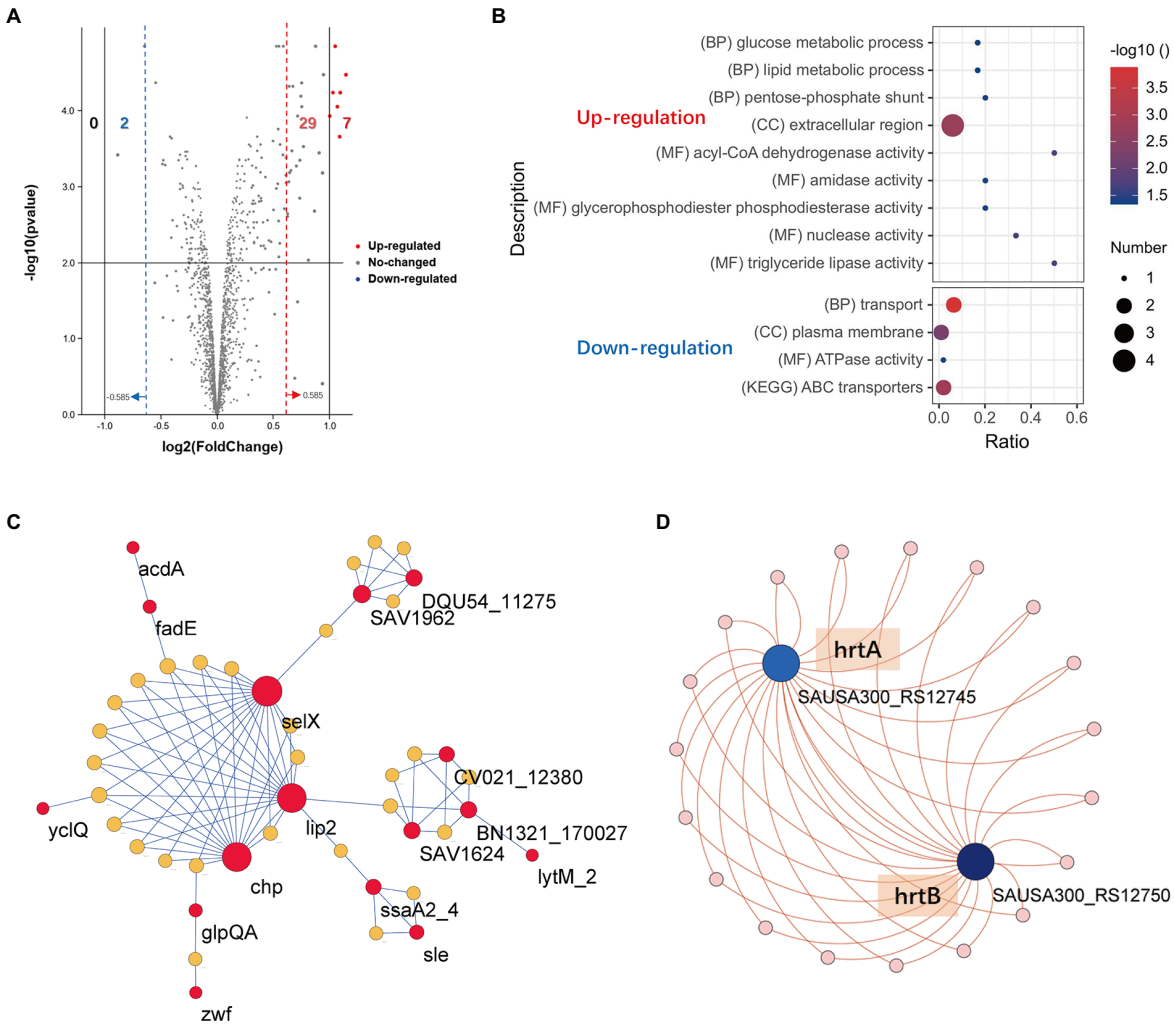
Proteomics profile change of *S. aureus* treated with TF. **(A)** Volcano plot visualizing the DEPs. The vertical lines divide the fold-change values. The right (red) vertical line corresponds to a change in log_2_FC > 0.585, while the left (blue) vertical line corresponds to a change in log_2_FC < −0.585. The horizontal line is corresponds to a  − log10 *p* value of 0.05. **(B)** Functional enrichment analysis of the upregulated and downregulated DEPs. **(C)** The PPI network of the 16 upregulated DEPs and 28 associated candidate genes created by the StaphNet web server. **(D)** The PPI network of the 2 downregulated DEPs and 19 related candidate genes created by the StaphNet web server. The interactions between proteins and the candidate gene symbols are shown in Supplementary Table S1.

We next sought to explore the effect of TF on the posttranscriptional landscape of *Staphylococcus aureus* based on TMT-labeled proteomics. We set the cutoff criteria to |logFC| > 0.585 (fold change >1.5) with a *p* value <0.05 to screen the DEPs and obtained 38 differentially expressed proteins (DEPs) in total, including 36 upregulated genes and 2 downregulated genes (Supplementary Table S2). Figure 2A shows the DEPs using a volcano plot. GO and KEGG pathway enrichment analyses were performed by the StaphNet web server. A total of 19 upregulated DEPs (including 6 toxin-related genes: *nuc*, *coa*, *selX*, *lip2*, *hysA* and *chp*) and 2 downregulated DEPs were matched with the StaphNet ID. The *nuc* gene encodes thermostable nuclease (TNase), which is unique to *S. aureus* and can hydrolyze host cell nucleic acids (Madison et al., 1983). The *coa* gene encodes *S. aureus* coagulase (Coa), an important virulence factor that protects *S. aureus* from the host’s immune system (Mcadow et al., 2012); the *selX* gene encodes staphylococcal enterotoxin-like proteins (SEs), which have superantigen activity and can also bind to neutrophils to inhibit their phagocytosis (Tuffs et al., 2017); the *lip2* gene encodes a fatty acid-modifying enzyme called glyceride hydrolase, which inactivates the bactericidal lipids secreted by the host at the abscess site, thereby promoting the growth of staphylococcal cells (Shryock et al., 1992); the *hysA* gene encodes hyaluronidase, which plays a crucial role in the early stages of subcutaneous infection by degrading hyaluronic acid in the extracellular matrix (ECM) of human tissues (Ibberson et al., 2016); and the *chp* gene encodes chemotaxis inhibitory protein of *S. aureus* (CHIPS), which is a secreted protein that specifically inhibits neutrophil and monocyte responses to fLMP and C5a mainly in the early stage of infection (Haas, and C., J.C., 2004).

Pathway-Centric search results showed that upregulated DEPs were enriched in the glucose metabolic process, lipid metabolic process and pentose-phosphate shunt, and 2 downregulated DEPs (hrtA and hrtB) were mainly involved in the transport process and ABC transporter pathway (Figure 2B). Network analysis revealed that 16 upregulated DEPs and 2 downregulated DEPs needed 28 and 19 candidate genes provided by StaphNet, respectively, to build minimal protein–protein interactive networks (PPI network) and displayed by the Cytoscape. As we observed, a total of 4 toxin-related genes (*lip2*, *selX*, *chp* and *hysA*) were involved in the construction of upregulated networks, and their activation increased the infectivity of *S. aureus*, but their dominant nodes remained in a stable expression state. The activation of lytm and inactivation of hrtA and hrtB may contribute to the inhibition of *S. aureus* proliferation (Figures 2C,D).

### RT–qPCR for validating proteomics results

**Figure 3:**
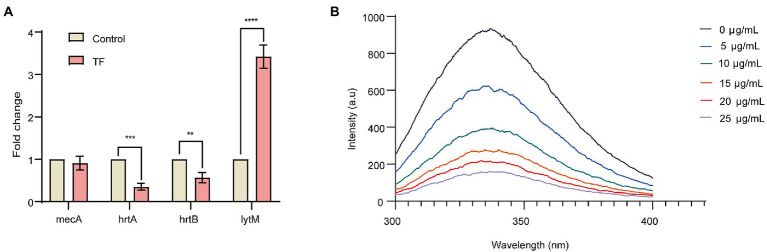
Relative expression analysis of candidate genes by qRT–PCR and binding of TF to PBP2a allosteric site. **(A)** Relative expression analysis of candidate genes by qRT–PCR. The RQ values on the Y-axis show fold-changes in mRNA expression relative to the control group. All values shown here are averages (±standard deviation) from three independent samples. The two-tailed Student’s t test was used for comparisons between groups. **p < 0.01, ***p < 0.001, ****p < 0.0001. **(B)** Emission scans of PBP2a intrinsic fluorescence with excitation at 280 nm. TF was titrated into give the final concentrations shown.

Based on the results of the interaction network of differentially expressed proteins, we selected 4 DEPs with the potential to inhibit the proliferation of *S. aureus* to analyze their transcription level changes by RT–qPCR. As illustrated in Figure 3A, when *S. aureus* USA300 was treated with 64 μg/mL TF, the *lytM* gene was upregulated, and the transcription levels of the *hrtA* and *hrtB* genes were downregulated. In addition, there was no difference in the transcription level of the *mecA* gene encoding the PBP2a protein, which was consistent with the proteomics results. These results also further reveal accuracy of the proteomics results.

### TF binds to the PBP2a allosteric site

The binding of TF to the PBP2a allosteric site was further investigated, and we measured the binding of the allosteric site by intrinsic fluorescence quenching of the purified PBP2a, which was covalently adjusted inside the active site using the antibiotic oxacillin (Bouley et al., 2015; Zhang et al., 2022). As shown in Figure 3B, with the increase of TF content, the fluorescence intensity of PBP2a allosteric site gradually decreased, and the calculated *K_d_* value was 1.87 ± 1.0 μg/mL, indicating that TF has a strong direct binding effect with PBP2a allosteric site.

### Evaluation of the synergistic effect of TF in combination with β-lactam antibiotics

To confirm the hypothesis that TF might enhance the affinity of PBP2a for β-lactam antibiotics by binding to the allosteric site to induce the opening of the active site based on the docking results, checkerboard assays were conducted to assess the synergistic effect of TF in combination with β-lactam antibiotics. The MIC of TF against USA300 was 1,024 μg/mL, and the MICs of β-lactam antibiotics against USA300 are presented in Table 2. The results showed that TF and several β-lactam antibiotics (ceftiofur, cefoxitin, latamoxef, ceftazidime, cefepime, oxacillin, ampicillin, and penicillin G) had synergistic antibacterial effects on *S. aureus* USA300, and the FICI ranged from 0.1875 to 0.3125. TF and ceftaroline fosamil had no synergistic effect on *S. aureus* USA300, and the FICI was 1.0625. Among the β-lactam antibiotics, the synergistic effect of TF in combination with ceftiofur was the most prominent, and the FICI was as low as 0.1875.

**Table 2:** Antibacterial effect of TF combined with β-lactam antibiotics against *S. aureus.*

Antibiotic	MIC _antibiotic_ (μg/mL)	FIC	MIC _TF_ (μg/mL)	FIC	FICI
Ceftiofur	8	0.125	>1,024	0.0625	0.1875
Cefoxitin	16	0.25	>1,024	0.0625	0.3125
Latamoxef	8	0.25	>1,024	0.0625	0.3125
Ceftazidime	128	0.25	>1,024	0.0625	0.3125
Cefepime	16	0.25	>1,024	0.0625	0.3125
Oxacillin	8	0.25	>1,024	0.0625	0.3125
Ceftaroline	8	1	>1,024	0.0625	1.0625
Ampicillin	512	0.25	>1,024	0.0625	0.3125
Penicillin G	256	0.25	>1,024	0.0625	0.3125

### Synergistic effect of TF and ceftiofur on MRSA clinical isolates

Given the significant synergistic effect of TF and ceftiofur against *S. aureus* USA300, we further investigated the synergistic effects of TF combined with ceftiofur on MRSA clinical isolates. As presented in Table 3, the MIC range of ceftiofur to MRSA clinical isolates was 32–64 μg/mL. However, the addition of TF led to a remarkable decrease in the MICs of ceftiofur against MRSA clinical isolates. The FICI of TF combined with ceftiofur on MRSA clinical isolates ranged from 0.125 to 0.3125.

**Table 3:** Synergistic antibacterial effect of TF and ceftiofur against MRSA clinical isolates.

Strain	MIC _antibiotic_ (μg/mL)	FIC	MIC _TF_ (μg/mL)	FIC	FICI
SA28	32	0.0625	>1,024	0.0625	0.125
SA34	64	0.125	>1,024	0.0625	0.1875
SA37	64	0.25	>1,024	0.0625	0.3125
SA1B2B	32	0.125	>1,024	0.0625	0.1875
SA1B3B	64	0.0625	>1,024	0.0625	0.125
SA1B5B	32	0.125	>1,024	0.0625	0.1875
SA1B3G	32	0.25	>1,024	0.0625	0.3125
SA1Z3G	64	0.0625	>1,024	0.0625	0.125

**Figure 4:**
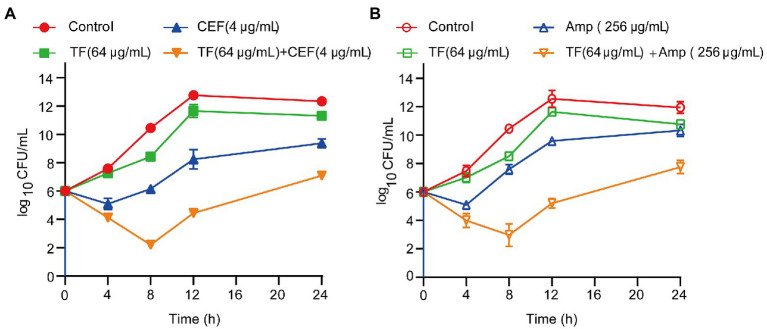
Time-killing kinetics of TF in synergistic combination with an antibiotic (CEF or Amp) against *S. aureus* USA300. **(A)** Time-killing kinetics of TF combined with CEF against *S. aureus* USA300. **(B)** Time-killing kinetics of TF combined with Amp against *S. aureus* USA300. Data represent 3 independent experiments ±SD.

The synergistic effects of TF in combination with two antibiotics (ceftiofur and ampicillin) against USA300 were further assessed by the time-kill curve assay. The growth curves of *S. aureus* USA300 showed that supplementation of TF (64 μg/mL) with 1/2 MIC ceftiofur led to a remarkable decrease in the growth of *S. aureus* USA300 within 4 h of culture, and the CFU reached the lowest point after incubation for 8 h (Figure 4A). Similar results were observed when TF (64 μg/mL) was combined with 1/2 MIC ampicillin against *S. aureus* USA300 (Figure 4B). These results revealed that TF combined with ceftiofur or ampicillin can significantly inhibit the early growth of bacteria. The combination of TF with ceftiofur or ampicillin caused more than 2 log_10_ CFU/mL reductions in *S. aureus* USA300 compared with the two antibiotics alone.

### Protective effect of TF combined with ceftiofur on pneumonia induced by *Staphylococcus aureus* USA300

**Figure 5:**
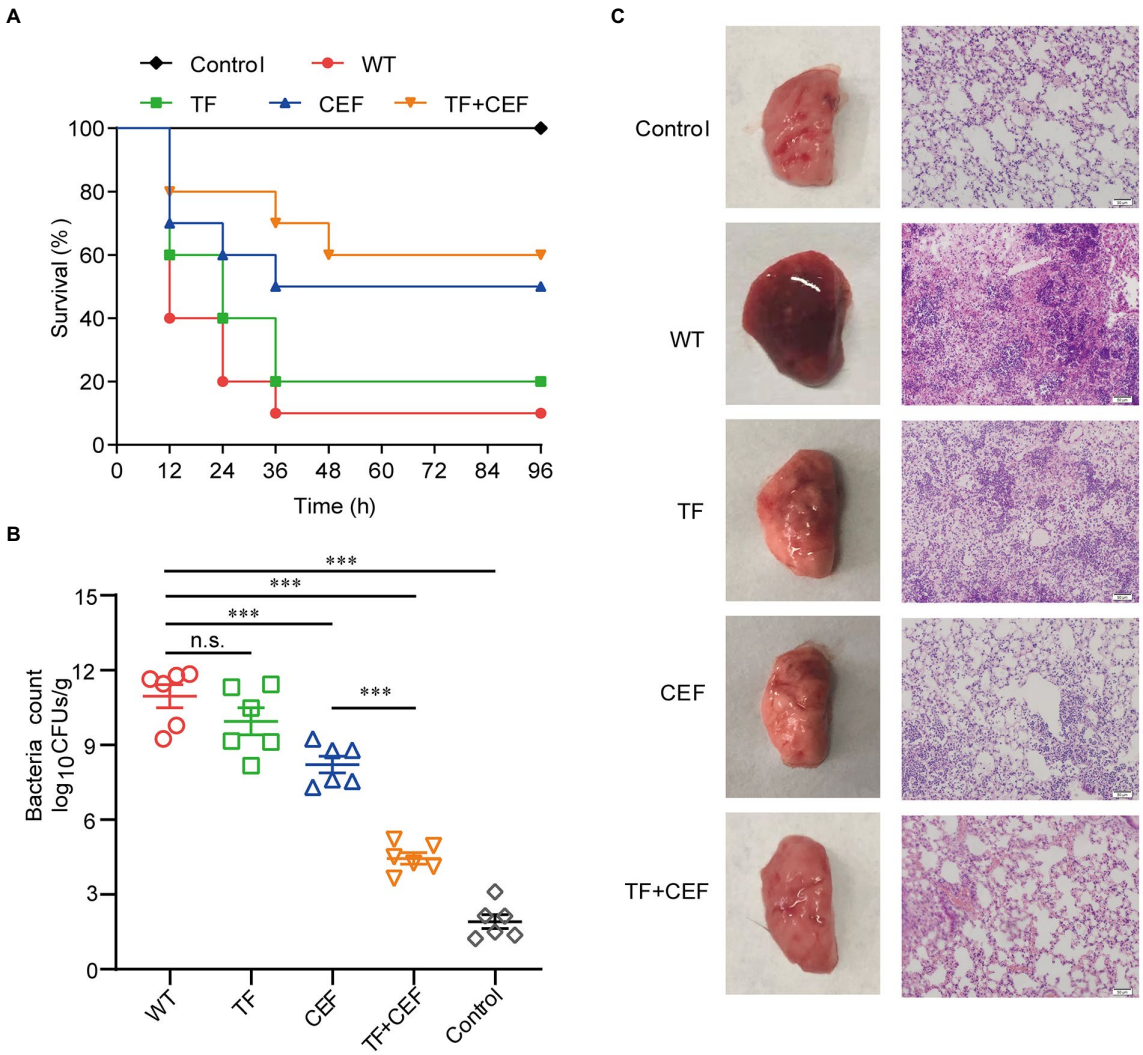
The effects of combination therapy. **(A)** Survival rate of mice at 96 h (*n* = 10). **(B)** Bacterial load counts in the lung tissues (*n* = 6). **(C)** Pathological changes and histopathology analysis of lung tissues. Scale bar = 50 μM. Data are presented as mean**±**SD **(n=3)**, n.s., no significant difference; ***p<0.001.

The most efficient drug combination (TF in combination with ceftiofur) was further validated *in vitro* by the therapeutic effect on the pneumonia model. Eight-week-old mice in each group were inoculated intranasally (i.n.) with a lethal dose of USA300 (4 × 10^8^ CFU/per mouse) and treated with TF and ceftiofur every 12 h. In addition, a single dose of TF or ceftiofur was administered in the same way as for the control group. As shown in Figure 5A, within 96 h of challenge with *S. aureus* USA300, only 10% of the mice survived, compared to 40% in the ceftiofur-treated group. Importantly, the combined treatment of TF (40 mg/kg) and ceftiofur (100 mg/kg) significantly increased the survival rate of mice to 60% (*p* < 0.001), especially in the early stage of infection.

The pulmonary bacterial load in the infection group was 11.58 ± 1.19 log_10_ CFU/g (Figure 5B). After treatment with ceftiofur, the bacterial load decreased to 7.6104 ± 0.69 log_10_ CFU/g, while the bacterial load in the combined group was only 4.26 ± 1.29 log_10_ CFU/g, indicating that TF combined with ceftiofur could significantly reduce the pulmonary invasion of *S. aureus* (*p* < 0.001).

The pathological changes in the lung tissue were further evaluated. No pathological changes were found in the lungs of the control group. The untreated infected group had some degree of acute injury, manifested as interstitial hyperemia and edema, with obvious inflammatory cell infiltration and hemorrhage in the alveolar space. However, TF combined with ceftiofur significantly alleviated the infiltration of inflammatory cells in the alveolar space, and the alveolar structure was relatively intact compared with that in the untreated infected group (Figure 5C). Thus, we concluded that the combination of TF and ceftiofur was significantly protective against pneumonia caused by lethal USA300.

## Discussion

It has been 80 years since the discovery and initiation of large-scale use of β-lactam antibiotics, and their consumption still ranks first among all antibiotics due to their broad-spectrum, nontoxic, and bactericidal effects and oral availability (Öztürk et al., 2015). However, their usefulness has been compromised by the development of resistance (Chambers and Deleo, 2009). Finding effective ways to deal with the drug resistance crisis against pathogens, especially MRSA, is still a huge challenge for humanity.

β-Lactam antibiotics inhibit transpeptidation through binding to PBPs on peptidoglycan strands. This hinders the synthesis of the cell wall, which eventually causes death of the bacteria (Kohanski et al., 2010). However, the active site of PBP2a in MRSA cannot be inhibited by β-lactam antibiotics, as it exists in a closed conformation with the active site serine deeply in a tight groove (Lim et al., 2002) and Novy et al., (2011). The possibility of an allosteric site in PBP2a was first reported in Cosimo et al. (2005). The crystal structures of PBP2a with ceftaroline molecules reported by Otero et al. confirmed the new drug resistance mechanism of PBP2a (Otero et al., 2013). On this basis, an allosteric mechanism was proposed: when the allosteric position was occupied by the natural substrate (or a ligand very similar to it), PBP2a underwent a conformational change, finally exposing the active center of the reaction. In our molecular docking results, TF showed stronger interactions at allosteric sites of PBP2a than at it active site, indicating a great potential to be a natural substrate that could occupy the allosteric site, leading to a β-lactam-accessible open active site.

The combination of natural products and antibiotics is widely regarded as a potential strategy to not only control bacterial proliferation effectively in the early stage of infection but also reduce the use of drugs and avoid the development of drug resistance (Boyd and Nailor, 2011; Tamma et al., 2012; Traugott et al., 2012). The significant advantages of antibiotic combinations make them widely used in both static (He et al., 2012) and dynamic models (Bergen et al., 2011) to combat multidrug-resistant pathogens. Our molecular docking results implied the possibility for TF to act as an allosteric site regulator of PBP2a, thereby restoring the sensitivity of MRSA to β-lactams.

Subsequent experiments confirmed this hypothesis. We found that TF combined with 8 kinds of β-lactam antibiotics had an obvious synergistic effect against *S. aureus* USA300. Among them, the combinations of TF with ceftiofur exhibited the strongest synergistic interactions against *S. aureus* USA300 and MRSA clinical isolates. The killing curve again proved the combined antibacterial effect *in vitro*. The *in vivo* results of the mouse pneumonia model were consistent with the above *in vitro* results. Another promising fact is that TF has no reported biohazards and no effect on the growth of USA300 (MIC>1,024 μg/mL). The high potency and lack of antibacterial activity of TF suggest that this compound is a promising candidate for use in combination with β-lactams against MRSA while reducing the occurrence of drug resistance to a certain extent.

The molecular docking results showed that in addition to the allosteric site of PBP2a, TF could also dock to the active site (the docking score at the active site was −5.360 kcal/mol). Subsequently, fluorescence quenching also further confirmed the ability of TF to bind to the PBP2a allosteric site. To further explore the effect of TF on MRSA, we used TMT proteomics analysis to observe the changes in the proteomic landscape of *S. aureus* after treatment with TF. TMT proteomics analysis has the advantages of being high throughput and having high sensitivity and good repeatability, which makes it an important tool to elucidate the mechanism of bacterial resistance and sensitivity to antibiotics. This extends our understanding of microbial behaviors under different environmental conditions or stimuli. In our TMT proteomics analysis results, the small number of differentially expressed proteins indicated that TF appeared to have very little effect on *S. aureus*.

Although the number of abnormally expressed genes was small in our proteomics results, the genes involved still require attention. TF activated 6 toxic proteins. In addition, Figure 2C shows that *lip2*, *selX* and *chp* formed a subnetwork, which possibly enabled TF to enhance the infectivity of *S. aureus*. However, activation of LytM and inactivation of ABC transporters seemed more likely to contribute to the inhibition of proliferation. LytM is an autolysin produced by *S. aureus* (Ramadurai et al., 1999). When the LytM protein is secreted out of the cell, it can specifically hydrolyze the peptide bond between the peptidoglycan glycine and glycine of the staphylococcal cell wall so that the bacteria are lysed. Although it has a low bactericidal capacity, fusing LytM to the cell wall binding domain increases its anti-staphylococcal activity by approximately 540-fold. Thus, it is comparable to many phage lysins that are still in the clinical development stage (Ercoli et al., 2015; Osipovitch and Griswold, 2015). ATP binding cassette (ABC) transporters are the largest class in the bacterial multidrug efflux transporter family, and they can mediate bacterial multidrug resistance and mainly rely on the hydrolysis of ATP to provide energy for substrate transport (Chang, 2003). HrtAB is an ABC-type transporter found in recent studies of *S. aureus* that is thought to protect the bacteria from damage in the presence of high concentrations of hemin (Bibb and Schmitt, 2010). The HrtAB system consists of an ATP enzyme (HrtA) and a membrane permeable enzyme (HrtB), which is hypothesized to export excess heme directly from the cytosol or remove toxic metabolites formed by heme accumulation (Stauff et al., 2007). In our study, TF appeared to trigger two opposing effects on *S. aureus*, although the effects were relatively weak. Based on the results of the current study, which effect is dominant is unclear.

In addition, we only verified the antibacterial effect of theaflavin with β-lactams antibiotics against the standard strain USA300 without involving the clinical isolates, which has some limitations. Theaflavins act as an allosteric agent of PBP2a, which is present in all MRSA. Thus, the combination of theaflavin and β-lactam antibiotics should have similar synergistic antibacterial effects against MRSA clinical strains, but further validation is needed.

In conclusion, the combination of TF and ceftiofur exhibited an excellent synergistic effect against MRSA both *in vitro* and *in vivo* and was superior to that of TF or ceftiofur alone. Molecular docking and proteomic results suggest that TF is highly likely to act as an allosteric agent of PBP2a, opening the active site of β-lactam. The combined application of TF and β-lactam antibiotics provides a new strategy for the treatment of MRSA infection.

## Data availability statement

The mass spectrometry proteomics data have been deposited to the ProteomeXchange Consortium via the PRIDE partner repository (https://www.ebi.ac.uk/pride/archive) with the dataset identifier PXD037668.

## Ethics statement

The animal study was reviewed and approved by the Committee on Animal Care and Use of Jilin University.

## Author contributions

DW and HY conceived and designed the experiments. SG, LZ, YJ, and JL performed the experiments, HY and HX performed the molecular dynamics simulation. SG and LW prepared the original manuscript. LW revised the manuscript. All authors contributed to the article and approved the submitted version.

## Funding

This work was supported by the Science Foundation of Jilin Province, China (No. 20180101276JC) and the National Key Research and Development Program of China (No. 2018YFD0500300).

## Conflict of interest

The authors declare that the research was conducted in the absence of any commercial or financial relationships that could be construed as a potential conflict of interest.

## Publisher’s note

All claims expressed in this article are solely those of the authors and do not necessarily represent those of their affiliated organizations, or those of the publisher, the editors and the reviewers. Any product that may be evaluated in this article, or claim that may be made by its manufacturer, is not guaranteed or endorsed by the publisher.

## Footnotes

^1^ https://pubchem.ncbi.nlm.nih.gov/

^2^ https://www.inetbio.org/staphnet/
