# Supplementary material for: Molecular docking and proteomics reveals the synergistic antibacterial mechanism of theaflavin with β-lactam antibiotics against MRSA
Source: Front Microbiol. 2022 Nov 14;13:993430. doi: 10.3389/fmicb.2022.993430 (PMC9702817; doi:10.3389/fmicb.2022.993430)
Supplement: Supplementary file 1 [file Table_1.DOCX]

**Table S1 | Oligonucleotide sequences**

| Primer | Sequence |
| --- | --- |
| *16S rRNA*-F | TCCGGAATTATTGGGCGTAA |
| *16S rRNA*-R | CCACTTTCCTCTTCTGCACTCA |
| *mecA*-F | GTTAGATTGGGATCATAGCGTCATT |
| *mecA*-R | GCCTAATCTCATATGTGTTCCTGTAT |
| *lytM*-F | TGGATCAGCAAGTAAAGCGACAGC |
| *lytM*-R | GCACCACCACCGTGATATTGTCC |
| *hrtA*-F | AAGAGGGATGCGAATGAAGA |
| *hrtA*-R | CACCTTGTGCGACAAAATCT |
| *hrtB*-F | CCTCCAATTGCTTCGATAGG |
| *hrtB*-R | TTATGATGCCGGTAACGATG |
